# Ethnomedicinal Evidence for Medicinal Plants of the Asteraceae Family Used by Tanzanians to Treat Various Ailments: A Review

**DOI:** 10.1155/bmri/4164568

**Published:** 2026-04-02

**Authors:** David Sylvester Kacholi

**Affiliations:** ^1^ Department of Biological Sciences, Dar es Salaam University College of Education, University of Dar es Salaam, Dar es Salaam, Tanzania, udsm.ac.tz

**Keywords:** ethnobotany, ethnomedicine, herbal remedies, indigenous knowledge, natural compounds, Tanzania

## Abstract

The Asteraceae family is amongst the most important groups of flowering plants, widely recognised for its ethnomedicinal relevance. This systematic review, conducted in line with the Preferred Reporting Items for Systematic Reviews and Meta‐Analyses (PRISMA) guidelines, synthesises evidence on the traditional medicinal uses of Asteraceae species in Tanzania. Data were compiled from multiple electronic databases, including but not limited to Web of Science, Scopus, Google Scholar, African Journals Online, PubMed and Wiley Online Library, and after rigorous screening, 31 studies from 16 regions were included. Findings reveal that 73 species are employed in traditional healing, predominantly for gastrointestinal disorders (35 species), dermatological conditions (20 species) and pain management (19 species). *Bidens pilosa*, *Ageratum conyzoides* and *Acmella caulirhiza* were the most frequently cited taxa. Herbs constituted the dominant life form (74%), with flavonoids (85.7%), terpenoids (54.8%) and phenolics (50.0%) identified as the principal bioactive compounds. Asteraceae MPs exhibit diverse pharmacological activities, including antibacterial, anti‐inflammatory, antioxidant and antidiabetic effects. Their therapeutic potential underscores the need for further phytochemical and pharmacological studies to validate bioactive compounds and their clinical relevance.

## 1. Introduction

Medicinal plants (MPs) are species that possess therapeutic properties or can exert beneficial pharmacological effects on the human or animal body [1, 2]. MPs have long been foundational to disease treatment in traditional medicine and indigenous practices worldwide. Many MPs have been used as medicinal agents for millennia in various traditional pharmacopoeias globally [3–8]. MPs are essential to conventional medicine, which encompasses practices, knowledge and beliefs that utilise plant‐, animal‐ and mineral‐based medicines and spiritual therapies. These methods, used alone or in combination, are used to diagnose, treat, and prevent illness or promote overall well‐being [9–11]. MPs are vital sources of natural products used in pharmaceutical drugs and health products [12]. Contemporary ethnopharmacological research emphasises the significance of MPs, as they play a crucial role in improving primary healthcare within local communities [13]. This is especially true in sub‐Saharan Africa, where rural and marginalised communities rely on traditional medicine as their primary healthcare resource.

Moreover, MPs represent a crucial link between traditional knowledge and modern sustainability objectives. Their significance in offering affordable and culturally appropriate healthcare directly supports Sustainable Development Goal (SDG) 3 (Good Health and Well‐Being), whilst their conservation preserves biodiversity and ecosystems, advancing SDG 15 (Life on Land). Simultaneously, sustainable harvesting practices contribute to SDG 12 (Responsible Consumption and Production) by decreasing reliance on synthetic chemicals and enhancing ecological balance. Collectively, these efforts underscore MPs as vital resources for improving human health, safeguarding the environment and promoting responsible resource management within the global sustainable development framework.

In low‐ and middle‐income countries, including Tanzania, nearly 80% of the population relies on MPs for their primary healthcare [14, 15]. Ethnobotanical studies conducted in Tanzania [16–19] have shown that MPs are extensively used in rural, peri‐urban and marginalised areas of the country, and their popularity continues to grow as natural alternatives to synthetic chemicals. The Asteraceae family, also known as Compositae, is a large and diverse group of flowering plants that includes over 1600 genera and about 23,000 species [20, 21]. These plants are found in diverse environments worldwide, ranging from rainforests to arid deserts. The significance of the Asteraceae family extends beyond its diversity [22]; it plays vital ecological roles, possesses ornamental value and offers numerous medicinal uses, making it a popular subject in both scientific research and horticulture [23–25]. The Asteraceae plant family has contributed several species to Tanzania’s traditional pharmacopoeia [26–28].

The Asteraceae family holds substantial cultural and economic importance globally, with its members serving as sources of therapeutic, food, ornamental, fodder and insecticidal products [20, 29, 30]. Many members of this family have been extensively studied for their bioactive chemical constituents [31–34] and pharmacological activities [20, 33, 35–37]. Assessing the phytochemical and pharmacological properties of specific Asteraceae species employed in traditional practices may facilitate the development of novel pharmaceuticals, functional foods and cosmetic products [38]. Although numerous secondary metabolites have been identified within this family, Asteraceae has been comparatively underrepresented in ethnopharmacological research [29, 39]. Therefore, this review was conducted to explore and document ethnomedicinal knowledge related to the Asteraceae family in Tanzania. By synthesising this information, it is aimed at identifying gaps in our understanding of the therapeutic potential of Asteraceae species. Additionally, the insights gained from this study will highlight promising areas for future ethnopharmacological research, encouraging further exploration and discovery within this field.

## 2. Materials and Methods

**Figure 1:**
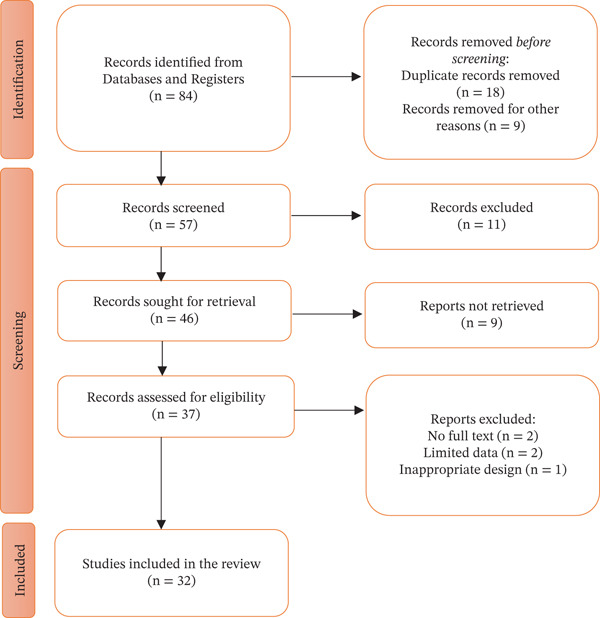
PRISMA flow diagram of the systematic review of medicinal Asteraceae plants of Tanzania.

This review analyses MPs from the Asteraceae family used to treat various human ailments in Tanzania, drawing on a range of sources. It provides a comprehensive compilation of MPs, along with an examination of traditional knowledge across different ethnic groups in the country. The review follows the Preferred Reporting Items for Systematic Reviews and Meta‐Analyses (PRISMA) guidelines (see Figure 1).

### 2.1. Literature Search Strategy

Ethnobotanical investigations documenting MPs of Asteraceae used by Tanzanians were identified through electronic databases, including PubMed, Wiley Online Library, Google Scholar, Scopus, African Journals Online (AJOL) and Web of Science. The main terminology used during the search includes ‘Asteraceae’, ‘Labiatae’, ‘traditional medicinal plants’, ‘medicinal plants’, ‘traditional knowledge’, ‘medicinal Asteraceae’, ‘medicinal Labiatae’, ‘ethnobotany’, ‘ethnobotanical study’, ‘indigenous knowledge’, ‘ethnomedicine’, ‘phytomedicine’, ‘United Republic of Tanzania’ and ‘Tanzania’. These search terms were employed both individually and in various combinations with Boolean operators such as ‘OR’ and ‘AND’. Data collected from each article comprised scientific names, life forms and plant parts utilised. The medicinal use categories were classified according to the Economic Botany Data Collection Standard. Scientific names of Asteraceae species were updated to reflect accepted names using the Plants of the World Online website (https://powo.science.kew.org/). This systematic approach ensures reliable results, enhances confidence in the research and provides a strong basis for future studies.

### 2.2. Inclusion Criteria

The inclusion criteria covered both published and unpublished ethnobotanical studies that highlight the use of MPs from the Asteraceae family by Tanzanians for managing various health conditions. This review focuses solely on original research articles in English from all administrative regions, aiming to provide valuable insights into the field.

### 2.3. Exclusion Criteria

Studies that lacked essential ethnobotanical details such as scientific names, parts used, methods or locations were excluded. Furthermore, the review excluded research on MPs for livestock, articles with limited access, those with minimal text or abstracts, non‐open‐access publications and studies conducted outside Tanzania.

### 2.4. Study Selection

The screening process involved reviewing titles and abstracts to establish eligibility. Subsequently, full‐text articles were evaluated according to predefined criteria. This systematic review included 32 studies from 16 administrative regions (see Figure 1).

### 2.5. Data Analysis

Descriptive statistical methods were employed to analyse the data, with results presented as frequences and percentagesand illustrated using tables, bar charts and pie charts.

## 3. Results and Discussion

### 3.1. MP Diversity

This review has identified 73 MPs from the Asteraceae family that Tanzanians frequently utilise for the management and treatment of various human ailments (Table 1). Recent ethnobotanical studies in Tanzania highlight the importance of exotic MPs in primary healthcare, as they offer therapeutic benefits, embody cultural heritage and ecological knowledge and support health and nutrition [68–70]. Elsewhere, such as in Brazil [71], India [72], Ethiopia [73], Nigeria [74] and South Africa [75], exotic MPs also form a significant part of the traditional pharmacopoeia. The exotic MPs have been mainly incorporated into herbal remedies due to their multiple uses.

**Table 1:** Asteraceae medicinal plants of Tanzania.

Species name	Local name	Region	Habitat	Part used	Ailment cured	Reference
*Acmella caulirhiza* Delile	Mtango (Pare), ighichánygárárimáte/ibhichángárárimáte (Kuria)	Kilimanjaro, Tanga, Mara	Herb	Leaf, root	Convulsions, broken limb, coughs, aphrodisiac, cold sores, toothache, malaria, stomatitis, chest pains, throat itching	[40, 41]
*Ageratum conyzoides* L.	Msendawazi (Nyamwezi), Omwigara, Omwiga, Katabtaba (Haya), Kundambara (Swahili), Matawana (Chagga), Waisébhó (Kuria), Entaha’nyenka/Akatabatabe (Nyambo)	Tabora, Kagera, Dar es Salaam, Kilimanjaro	Herb	Root, leaf, flower, whole plant	Asthma, coughs, cold, peptic ulcers, stomachache, constipation, wounds/cuts, fibroids, women with conceiving difficulties, cancer, lung disorders, stomach abscess, urinary tract infections	[40, 42–48]
*Aldama excelsa* (Willd.) E.E.Schill. & Panero	Eghetabhárári/ibhitabhárári (Kuria)	Mara	Herb	Leaf	Yellow fever	[47]
*Ambassa hochstetteri* (Sch.Bip. ex Hochst.) Steetz	Msolawake (Zigua), Mluka (Sambaa)	Tanga, Pwani	Shrub	Root, whole plant	Stomachaches, epilepsy	[40, 49]
*Artemisia afra* Jacq. ex Willd.	Fivi (Swahili), Enjanipus (Maasai)	Dar es Salaam, Dodoma, Arusha	Herb	Leaf, root	Malaria, headache	[46, 50, 51]
*Aspilia africana* (Pers.) C.D.Adams	Mvuti (Swahili)	Dar es Salaam	Herb	Whole plant	Skin rashes	[50]
*Aspilia mossambicensis* (Oliv.) Wild.	Kanyamuza, Kashenganzili (Haya), Suruwa (Swahili), Mgeregere (Zigua), Nyangaanyangaa (Sukuma)	Kagera, Pwani, Dar es Salaam, Tanga, Mwanza	Herb	Root, leaf	Convulsions, retarded growth in children, conjunctivitis	[40, 46, 52]
*Baccharoides hymenolepis* (A.Rich.) Isawumi, El‐Ghazaly & B.Nord.	Msungu (Luguru)	Morogoro	Herb	Root, leaf	Fever, diarrhoea, hernia, spleen enlargement	[18]
*Baccharoides lasiopus* (O.Hoffm.) H.Rob.	Itughutu (Pare), Mtugutu (Chagga), Mhasha (Sambaa)	Kilimanjaro, Tanga	Shrub	Root. leaf	Stomachaches, female sterility, to regulate menses, urinary tract infections	[40, 48, 49]
*Berkheya bipinnatifida* (Harv.) Roessle	Not specified	Pwani, Mara	Herb	Leaf	Menorrhagia	[53, 54]
*Bidens holstii* Sherff	Mbangwi (Hehe)	Iringa	Herb	Root	Coughs	[55]
*Bidens pilosa* L.	Nyaweza, Ndasa (Nywamwezi), Pwimbiji (Zaramo), Kimbara (Pare), Kakurana (Haya, Nyambo), Mshona nguo (Swahili), Vishikishio (Sukuma), iritótónimaísó/amatótónimaísó (Kuria), Mgorogombe (Ha), Enterepeni (Maasai), Lipuli (Hehe)	Morogoro, Tabora, Pwani, Kilimanjaro, Mwanza, Kagera, Dar es Salaam, Mara, Kigoma, Arusha, Iringa	Herb	Bark, leaf, flower, fruits, whole plant	Wounds, cuts, malaria, fever, headache, kidney problems, burns, ringworms, diarrhoea, blood clotting problem, haematopoietic, constipation, stomachaches, spleen enlargement, mouth oral infection, intestinal worms, anaemia, urinary tract infections	[18, 27, 40, 41, 46–48, 50–52, 56–63]
*Bidens taylorii* Sherff	Rwongera (Haya), Mgorogombe (Ha)	Kagera, Kigoma	Herb	Leaf, flower	Malaria, fever, cardiac palpitations, anaemia	[52, 61]
*Bidens whytei* Sherff	Mpangwe (Swahili), Mgorogombe (Ha)	Dar es Salaam, Kigoma	Herb	Leaf, root, flower	Anaemia	[50, 61]
*Blepharispermum zanguebaricum* Oliv. & Hiern	Chitandachaulu (Makonde), Mnenga (Zigua), Msekele (Zaramo)	Mtwara, Tanga, Pwani, Dar es Salaam	Shrub	Leaf	Menstruation, hernia, snakebite	[40]
*Bothriocline longipes* (Oliv. & Hiern) N.E.Br.	Endishai (Maasai)	Arusha	Herb	Root	Venereal diseases, colic, body pains	[51]
*Brachylaena huillensis* O.Hoffm.	Mhungwe (Zigua), Mkarambati (Swahili)	Pwani, Tanga	Tree	Leaf	Bilharzia and gonorrhoea	[40]
*Chromolaena odorata* (L.) R.M.King & H.Rob.	Irihémbúhémbú/amahémbúhémbú (Kuria)	Mara	Herb	Leaf	Urinary tract infections, abdominal pain	[41, 47]
*Chrysanthellum indicum* DC.	Irinyányámá/amanyányámá (Kuria)	Mara	Herb	Leaf	Purge the stomach	[47]
*Crassocephalum crepidioides* (Benth.) S.Moore	Eghetóóma/ibhitóóma (Kuria)	Mara	Herb	Leaf	Burning sores	[47]
*Crassocephalum montuosum* (S.Moore) Milne‐Redh.	Nyaluganza	Morogoro	Herb	Bark	Earache, headache, burn	[18, 27]
*Crassocephalum picridifolium* (DC.) S.Moore	Amabei (Iraqw’)	Manyara	Herb	Root	Boils	[64]
*Crassocephalum vitellinum* (Benth.) S.Moore	Ekishenda (Haya), Uwenge (Swahili)	Kagera, Dar es Salaam	Herb	Aerial parts, leaf, whole plant	Peptic ulcers, mouth sores, shingles or herpes zoster, urinary tract infections	[45, 48–50]
*Cyanthillium cinereum* (L.) H.Rob.	Ikibhuríá/ibhibhuríá (Kuria), Mbalikila (Hehe)	Mara, Iringa	Herb	Root	Abdominal pain, stomachaches, coughs	[47, 55]
*Dichrocephala integrifolia* (L.f.) Kuntze	Shinda kaya (Sambaa), Ibuza (Haya), Kikumbi (Pare)	Tanga, Kagera, Kilimanjaro	Herb	Leaf, whole plant	Stomach and mouth ulcers, fracture, bloody diarrhoea, eye infections	[40, 43, 44, 49]
*Dicoma anomala* Sond.	Karibekantu (Sukuma)	Mwanza	Herb	Root	Heartburn	[46]
*Emilia coccinea* (Sims) G.Don	Sunga (Hehe)	Mara, Iringa	Herb	Whole plant	Cancer, deworming	[55, 65, 66]
*Emilia discifolia* (Oliv.) C.Jeffrey	Imangwe (Ruri)	Mara	Herb	Whole plant	Syphilis	[65]
*Emilia fosbergii* Nicolson	Kanyorokazi (Sukuma)	Mwanza	Herb	Leaf	Lung problems	[46]
*Emilia javanica* (Burm.f.) C.B.Rob.	Kamuguze, Kiemba cha bwana (Zaramo)	Pwani	Herb	Leaf	Vomiting and diarrhoea	[40]
*Erigeron bonariensis* L.	Ikinyáitóróra/ibhinyáitóróra (Kuria)	Mara	Herb	Leaf	Urinary tract infections, yellow fever, mycosis	[41, 47]
*Erigeron canadensis* L.	Akamwisanga (Kerewe)	Mwanza	Herb	Whole plant	Snakebite	[67]
*Eschenbachia japonica* (Thunb.) J.Kost.	Mterezi (Vidunda)	Morogoro	Herb	Root	Female sterility	[40]
*Felicia grantii* (Oliv. & Hiern) Grau	Nyaseko‐indume (Jita)	Mara	Herb	Sap, root	Eye infections, stomach pains	[65]
*Galinsoga parviflora* Cav.	Esirwa (Maasai)	Arusha	Herb	Leaf	Wounds and body wash	[51]
*Gutenbergia polycephala* Oliv. & Hiern	Akatoma (Haya)	Kagera	Herb	Leaf	Prevent belching	[44]
*Gymnanthemum auriculiferum* (Hiern) Isawumi	Kishwiya (Haya)	Kagera	Shrub	Leaf	Febrile convulsions	[43]
*Gymnanthemum coloratum* (Willd.) H.Rob. & B.Kahn	Mhasahanda (Sambaa)	Tanga	Shrub	Root	Swellings	[49]
*Gymnanthemum myrianthum* (Hook.f.) H.Rob.	Mtugutu (Hehe)	Iringa	Shrub	Root	External boils	[55]
*Helianthus annuus* L.	Ufuta (Swahili)	Morogoro	Herb	Leaf	Chest pain, asthma	[18, 27]
*Helichrysum foetidum* (L.) Moench	Linusi (Hehe)	Iringa	Herb	Leaf	Pneumonia	[55]
*Helichrysum odoratissimum* (L.) Sweet	Emukutiyai (Maasai)	Arusha	Herb	Leaf, root	Wounds, cough, chest pain	[51]
*Helichrysum schimperi* (Sch.Bip. ex A.Rich.) Moeser	Lweza (Luguru)	Morogoro	Herb	Root	Stomachache, diarrhoea	[18, 27]
*Helichrysum setosum* Harv.	Msindiko (Chagga)	Kilimanjaro	Herb	Leaf	Epilepsy	[40]
*Jeffreycia hildebrandtii* (Vatke) H.Rob., S.C.Keeley & Skvarla	Muluka (Sambaa), Uswaswaki (Zigua)	Tanga, Pwani	Shrub	Root, leaf	Convulsions in children, mental illness	[40, 49]
*Jeffreycia usambarensis* (O.Hoffm.) H.Rob., S.C.Keeley & Skvarla	Lupasa (Hehe)	Iringa	Herb	Root	Tuberculosis, wound	[55]
*Jeffreycia zanzibarensis* (Less.) H.Rob., S.C.Keeley & Skvarla	Ntawatawa (Makonde)	Mtwara, Dar es Salaam	Shrub	Root	Psychiatric problems, chest diseases, spleen or kidney pains, hernia	[40]
*Launaea cornuta* (Hochst. ex Oliv. & Hiern)	Mchunga (Swahili), Mshunga (Sambaa), Kihawa (Luguru)	Tabora, Tanga, Morogoro, Pwani	Herb	Leaf	Diarrhoea, epilepsy, gonorrhoea, ascariasis, stomach pain, convulsions in children	[40, 49, 57]
*Linzia glabra* Steetz	Mukalikali (Nyamwezi), Mkutuku‐ngonda (Ngindo), Rushalila ya kansa (Sukuma), Afaxawi (Iraqw’)	Tabora, Ruvuma, Morogoro, Mwanza, Manyara	Herb	Root	Gonorrhoea, syphilis, bilharzia, amoebic dysentery, cancer, impotence, sexually transmitted infections	[16, 40, 46, 64]
*Lipotriche scandens* (Schumach. & Thonn.) Orchard	Byabarwoya (Haya)	Kagera	Herb	Leaf	Ulcers, wounds, lowering blood glucose	[43, 44]
*Microglossa pyrifolia* (Lam.) Kuntze	Kichuaghembe (Pare), Mfulufulu (Zaramo), Mlenga (Zigua), Omuhe, Mkuraiju (Haya)	Kilimanjaro, Pwani, Dar es Salaam	Shrub	Whole plant, leaf	Fever, heartburn, epilepsy, hernia, uterine prolapse, headache, cold, coughs, scalds, flu	[40, 43, 49]
*Microglossa pyrrhopappa* (Sch.Bip. ex A.Rich.) Agnew	Mshashu, Muuka (Sambaa), Rufugakande (Sukuma), Ankwey (Iraqw’)	Tanga, Mwanza, Manyara	Shrub	Root, leaf	Convulsions in children, malaria, persistent cough, frequent fevers	[46, 49, 64]
*Montanoa hibiscifolia* Benth.	Omosighóngóghe/imisighengoghe (Kuria)	Mara	Shrub	Leaf	Yellow fever	[47]
*Orbivestus cinerascens* (Sch.Bip.) H.Rob.	Mbalike (Sukuma)	Mara, Mwanza	Herb	Leaf	Skin infections, mental disorders	[65]
*Pluchea dioscoridis* (L.) DC.	Mnywenywe (Swahili, Zigua)	Tanga, Pwani	Shrub	Leaf, root, bark	Fever, backaches, skin diseases, colds, hernia, dysmenorrhoea, convulsions	[40, 49]
*Pseudoconyza viscosa* (Mill.) D’Arcy	Nyamgoha (Hehe)	Iringa	Herb	Leaf	Conjunctivitis, eye ache	[55]
*Psiadia punctulata* Vatke	Mpalaghasha (Sambaa, Zigua), Endimii (Maasai)	Tanga, Arusha	Shrub	Root, leaf	Epilepsy, abdominal pain, cough, asthma, fever, wounds due to burns	[49, 51]
*Silybum marianum* (L.) Gaertn.	Gidou (Swahili)	Dar es Salaam	Herb	Root	Liver diseases	[50]
*Solanecio angulatus* (Vahl) C.Jeffrey	Leza (Sambaa), Ihorohoro (Chagga)	Tanga, Kilimanjaro	Herb	Leaf	Peptic ulcers, malarial convulsions, cerebral malaria, nausea, body sores, diarrhoea, vomiting	[40, 49, 59]
*Solanecio cydoniifolius* (O.Hoffm.) C.Jeffrey	Hanamwiko (Luguru), Kikarabo, Eiraire (Haya)	Morogoro, Kagera	Herb	Leaf	Convulsions, ulcerations, wounds, swellings, stiff neck, emetic	[40, 43]
*Solanecio mannii* (Hook.f.) C.Jeffrey	Akagango‐akake (Haya), Ilenge (Pare)	Kagera, Kilimanjaro	Tree	Root, aerial parts	Febrile convulsions, stomachaches, abdominal pains, urinary tract infections, malaria, emetic	[40, 45, 47]
*Sonchus oleraceus* L.	Orunoko (Haya)	Kagera	Herb	Whole plant	Peptic ulcer disease	[66]
*Sonchus pinnatifidus* L.	Sungasunga (Luguru)	Morogoro	Herb	Root, leaf	Stomachaches, headache	[18, 27]
*Sonchus schweinfurthii* Oliv. & Hiern	Mchunga (Swahili)	Dar es Salaam	Herb	Leaf	Malaria, blood pressure, typhoid, diabetes, gonorrhoea	[50]
*Sphaeranthus steetzii* Oliv. & Hiern	Orkipirelekima (Maasai)	Arusha	Herb	Leaf, root	Menstrual pains, joint pain	[51]
*Tagetes minuta* L.	Nyashan (Barbaig)	Manyara	Herb	Leaf	Intestinal worms	[19]
*Tarchonanthus camphoratus* L.	Oleleshwa (Maasai)	Arusha	Shrub	Leaf, root, bark	Bedsores, skin irritations, toothache, cough	[51]
*Tithonia diversifolia* (Hemsl.) A.Gray	Maua (Swahili, Sukuma), irichonkíná/amachónkíná inchókíná/ichinchókíná (Kuria)	Mara, Mwanza	Herb	Leaf	Skin infections, stomach problems, urinary tract infections, eye problems	[47, 65]
*Tridax procumbens* L.	Mkakara (Swahili)	Mwanza	Herb	Leaf	Urinary tract infection, malaria	[46]
*Vernonia amygdalina* Del.	Musungu (Luguru), Mtugutu (Zigua), Mgorogombe (Ha)	Morogoro, Pwani, Kigoma	Shrub	Root, leaf	Fever, diarrhoea, malaria, diabetes mellitus, headache, joint pain, snakebite	[27, 40, 61]
*Vernonia galamensis* (Cass.) Less.	Iraiho (Chagga)	Kilimanjaro	Shrub	Leaf	Stomachaches	[40]
*Vernonia gigantea* (Walter) Trel.	Irirárárwé/amarárárwé (Kuria)	Mara	Shrub	Leaf, root	Dental pain, urinary tract infections, sharp abdominal pain	[47]
*Vernonia iodocalyx* O.Hoffm.	Kitugutu (Luguru)	Morogoro	Herb	Bark, leaf	Stomachache, diarrhoea, headache	[18, 27]

Of the recorded genera, 12 have at least two species, accounting for about 54.2% (i.e., 39 species) of the recorded MPs. *Emilia*, *Vernonia*, *Helichrysum*, *Crassocephalum* and *Bidens* are the genera with the most MPs (each comprising four species), followed by *Solanecio*, *Jeffreycia* and *Gymnanthemum* (each with three species), as well as *Sonchus*, *Microglossa*, *Aspilia*, *Erigeron* and *Baccharoides* (each with two species). Each of the remaining 33 genera contains a single species. Nonetheless, the genus with the most literature records is *Bidens* (19 records), followed by *Ageratum* and *Crassocephalum* (each with 8 records), *Microglossa* (6 records) and *Vernonia* (5 records) (Table 1). These genera are frequently cited in ethnobotanical studies because they are abundant, widely distributed and deeply integrated into local medicinal and cultural practices [76–80]. This prominence signals that future research should prioritise systematic pharmacological validation, conservation strategies and deeper sociocultural analysis of these genera.

**Figure 2:**
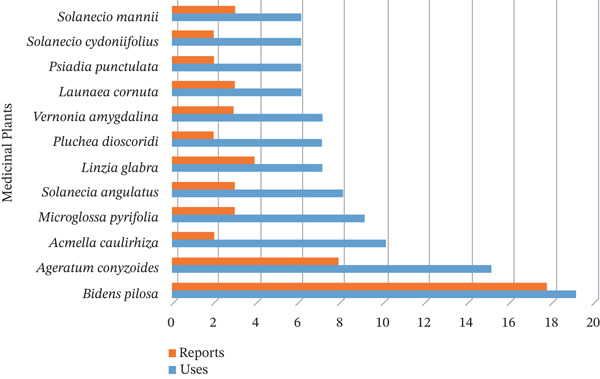
Number of medicinal uses and reports of the Asteraceae family.

Amongst the recorded MPs, *Bidens pilosa* (19), *Ageratum conyzoides* L. (15) and *Acmella caulirhiza* Delile (10) have the highest medicinal uses in the country, with the first two consistently documented in at least five independent sources of literature (Figure 2). *B. pilosa* is also reported in Zimbabwe to treat and manage stomach pains, anaemia, hypertension, oral thrush, and toothache, as well as for use during pregnancy [81, 82]; in Kenya [83], it is used to treat malaria; in Uganda [84–86], it is employed to treat paediatric diseases, malaria and wounds; and in South America [87], it is used as an antihypertensive and vasodilation agent. *A. conyzoides* is utilised in India [88] to address ailments such as respiratory tract infections, fever, wounds and inflammation; in Brazil [89] for hypolipidaemic use; and in Nigeria [90] for bleeding, measles and diabetes. Other MPs reported elsewhere include *Linzia glabra* Steetz, which is used for gonorrhoea, infertility in women, abdominal pains, burns and red eyes; as an abortifacient in Zimbabwe [39]; and as an antivenin in Kenya [91]. *Vernonia amygdalina* Delile is used in Uganda for treating malaria [86]. The analogies and discrepancies in the utilisation of these MPs (Figure 2) across nations are essential for analysing their ethnopharmacological and toxicity properties. Therefore, understanding their diverse applications enhances knowledge of cultural practices and emphasises the medicinal potential of each MP.

### 3.2. Distribution of Medicinal Asteraceae Plants Across the Country

**Figure 3:**
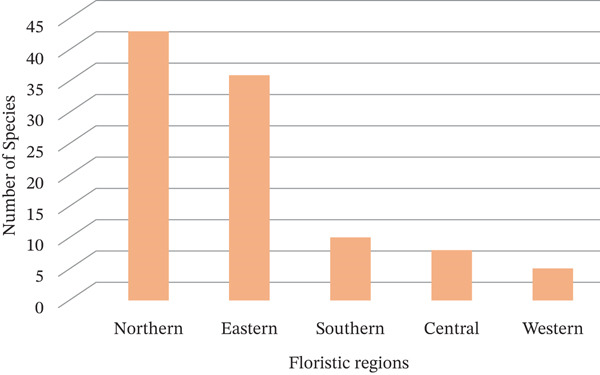
Distribution of Asteraceae medicinal plants in Tanzania.

The Northern floristic region of Tanzania recorded the highest proportion of Asteraceae MPs, accounting for 59.7% of all species documented in this review (Figure 3). This was followed by the Eastern region (50.0%), another Northern subset (42 species, 84.0%), the Southern region (13.9%), the Central region (11.1%) and the Western region (6.9%). The observed distribution highlights a pronounced concentration of collection efforts in the Northern and Eastern regions, corroborating the assertion by Kacholi and Amir [16, 17, 42] that the Western and Central floristic regions remain undercollected. This imbalance reflects historical patterns in botanical exploration, where researchers have favoured regions considered more accessible or biologically diverse. In some cases, collectors enrich herbarium specimens with ethnobotanical annotations, offering valuable insights into the cultural dimensions of plant use. Ethnobotanical research thus serves as a critical bridge between indigenous knowledge and scientific inquiry, reinforcing biocultural conservation [92]. The documentation of MP applications tends to expand alongside intensified collection efforts and the systematic integration of ethnobotanical perspectives. Nevertheless, the uneven distribution of studies limits the comprehensiveness of available evidence, particularly from underrepresented regions. Future research should therefore prioritise expanding coverage in Western and Central Tanzania.

### 3.3. Growth Habit and Part Used

**Figure 4:**
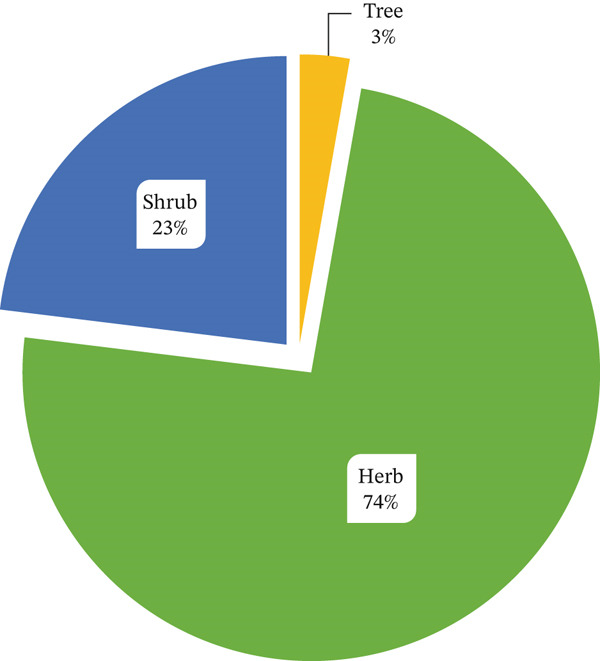
Growth habit of medicinal Asteraceae plants from Tanzania.

**Figure 5:**
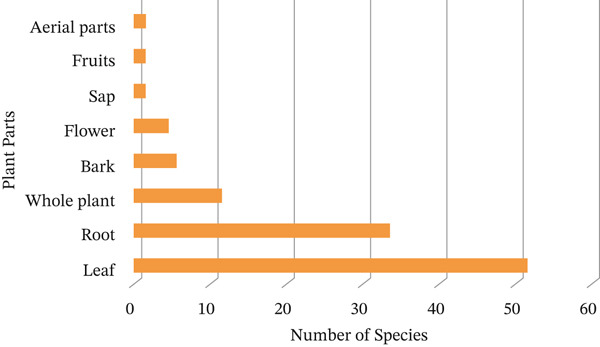
Plant parts utilised in herbal remedy formulation.

Herbs constitute 74% of the primary sources of Asteraceae MPs in Tanzania, followed by shrubs at 23% and trees at 3% (Figure 4). The plant parts used in herbal remedies include roots, leaves, fruits, bark and whole plants (Figure 5). Amongst these, leaves account for 70.8% of the MPs, whilst roots constitute 45.8%, making them the most commonly used parts. Similar findings have also been reported in South Africa concerning this plant family [39]. Harvesting roots from herbaceous plants for medicinal purposes raises serious sustainability concerns, endangering the survival of key species in traditional medicine [93, 94]. This issue is particularly prominent within the family, where many herbs are at risk of overexploitation.

In addition to overharvesting, climate factors such as droughts, shifting rainfall and rising temperatures further reduce the availability of MPs by disrupting growth cycles and regeneration [95]. To mitigate these pressures, sustainable practices are vital, favouring aerial parts over roots, rotating harvests to allow recovery and involving communities in monitoring. Cultivating high‐demand MPs in home gardens and agroforestry systems, whilst blending traditional knowledge with modern conservation [17, 93], will help secure the long‐term availability of Asteraceae MPs.

Despite ongoing conservation efforts in Tanzania, significant gaps remain in data on the conservation status of Asteraceae MPs within the country. This situation contrasts markedly with the more comprehensive research conducted in other nations, where information on plant conservation is more accessible and systematically documented. Therefore, promoting the sustainable utilisation and management of popular Asteraceae MPs, which are of great importance to local communities, is of utmost priority.

### 3.4. Ailment Categories

Ailments in this review are classified into 26 broad categories (Table 2), which are not strictly exclusive. Multipurpose MPs were included in all relevant categories to reflect their diverse uses. Within this framework, MPs belonging to the family Asteraceae are most frequently employed in the management of gastrointestinal conditions (47.9%), followed by dermatological disorders (27.3%), pain‐related ailments (26.0%), malaria and fever (21.9%), respiratory disorders (19.1%) and neurological conditions (17.8%) (Table 2). These findings are consistent with ethnobotanical surveys conducted in Zimbabwe, which similarly highlight the therapeutic prominence of Asteraceae species in addressing gastrointestinal and respiratory disorders, reproductive health complications and sexually transmitted infections. Notably, these conditions correspond to several of the leading causes of mortality in the country, underscoring the epidemiological significance of traditional medicinal practices and the potential role of Asteraceae taxa in primary healthcare strategies [96, 97]. Moreover, poor solid waste management has been identified as a critical determinant of gastrointestinal and respiratory infections in Tanzania [98] and other African countries, like Ethiopia [99] and Nigeria [100], a challenge that has progressively intensified over the past decades. Comparable burdens are evident across the continent, with dysentery and diarrhoea remaining persistent threats in Mozambique [101] and South Africa [102, 103]. Taken together, these findings highlight the intersection of environmental governance and public health, underscoring the need for integrated policy frameworks that strengthen waste management infrastructure, promote preventive health strategies and reduce the incidence of avoidable disease across the country and the continent.

**Table 2:** Major ailment categories.

Ailment category	Number of species
Gastrointestinal disorders	35
Dermatological disorders	20
Pains (Back, feet, chest, headache)	19
Malaria and fever	16
Respiratory disorders	14
Neurological problems	13
Urinary transmitted infections	10
Gynaecological disorders	7
Sexually transmitted infections	7
Hernia	5
Ophthalmological disorders	5
Antivenin	3
Reproductive disorders for men	3
Anaemia	3
Dental problems	3
Nephrological disorders	3
Cancer	3
Otolaryngological disorders	3
Blood pressure disorder	2
Bilharzia	2
Mental problems	2
Splenomegaly	2
Cardiac disorders	1
Diabetes mellitus	1
Hepatological disorders	1
Orthopaedic disorders	1

### 3.5. Photochemical and Pharmacological Evidence

The medicinal Asteraceae plants are rich in various chemical constituents (Table 3). Most of the documented MPs (Table 3) are characterised by flavonoids (85.7%), followed by terpenoids (54.8%), phenolics (50.0%), tannins (47.6%), alkaloids (40.5%), saponins (40.5%), glycosides (38.1%), steroids (28.5%) and quinones (23.8%). Conversely, most documented MPs exhibit several proven pharmacological activities (Table 3), such as antibacterial (57.1%), anti‐inflammatory (54.8%), antioxidant (54.8%), antimicrobial (40.5%), antifungal (33.3%), antimalarial/antiplasmodial (31.0%), hepatoprotective (21.4%) and antidiabetic (19.0%) (Table 3). The findings of this review corroborate those of Maroyi [39], who identified flavonoids, terpenoids, tannins and saponins as the predominant phytochemical constituents in Asteraceae MPs of Zimbabwe, which are likewise reported to occur in highly therapeutic MPs within the family in Egypt [183]. Despite this family’s richness in secondary metabolites, few ethnopharmacological studies have been conducted on this family [29]. Additionally, a study by Rolnik and Olas [20] supports the findings of this review, showing that members of this family exhibit anti‐inflammatory, antimicrobial, antioxidant and hepatoprotective properties. Furthermore, some of the documented MPs in this study have significant commercial value in both local and international markets [20, 46, 101, 184–186]. These MPs include but are not limited to *A. conyzoides* L., *Artemisia afra* Jacq. ex Willd., *Aspilia mossambicensis* (Oliv.) Wild., *B. pilosa* L., *Dicoma anomala* Sond., *Emilia fosbergii* Nicolson, *L. glabra* Steetz, *Microglossa pyrrhopappa* (Sch.Bip. ex A.Rich.) Agnew, *Tridax procumbens* L. and *Gymnanthemum coloratum* (Willd.) H.Rob. & B.Kahn.

**Table 3:** Phytochemical constituents and pharmacological activities of some Asteraceae medicinal plants.

Species name	Phytochemical constituents	Pharmacological activities	Reference
*Acmella caulirhiza* Delile	Spilanthol, flavonoids, phenols, vanillic acid, scopoletin, tannins, terpenoids, steroids and fatty acids	Antioxidant, antibacterial, antitumor and anti‐inflammatory	[104–106]
*Ageratum conyzoides* L.	Alkaloids, terpenes, chromenes, flavonoids, sterols, coumarins, benzofurans and terpenoids	Antidiabetic, antimicrobial, antifungal, anticancer, antiprotozoal, antioxidant, anti‐inflammatory, analgesic, spasmolytic, anthelmintic, antibacterial, nematicidal, wound healing and radioprotective	[107, 108]
*Artemisia afra* Jacq. ex Willd.	Alkaloids, cardiac glycosides, flavonoids, saponins, essential oils, phenols, tannins and terpenoids	Antimalarial, antimicrobial, antioxidant, antibacterial, antiviral, antiaflatoxin, antihistaminic, bronchodilator, narcotic analgesic and anti‐inflammatory	[109–116]
*Aspilia africana* (Pers.) C.D.Adams	Alkaloids, saponins, flavonoids, tannins, phenols, terpenoids, sterols and glycosides	Antimicrobial and anti‐inflammatory	[117, 118]
*Aspilia mossambicensis* (Oliv.) Wild.	Flavonoids, alkaloids, saponins, steroids and anthraquinones	Antifungal, antibiotic and antioxidant	[119–121]
*Baccharoides lasiopus* (O.Hoffm.) H.Rob.	Cardiac glycosides, coumarins, flavonoids, steroids, phenolics, reducing sugars, saponins, tannins, terpenoids, elemanolides, alkaloids, anthraquinones and xanthines	Anthelmintic, antibacterial, antifungal, antiviral, larvicidal, antihyperglycaemic, antiplasmodial, antiprotozoal, haematological, antimalarial, hepatoprotective, hepatotoxicity and cytotoxicity	[122]
*Bidens pilosa* L.	Flavonoids, fatty acids, phenolic acids, cardiac glycosides, terpenoids, phytosterols and chalcones	Antioxidant, antibacterial, anticancer, cytotoxic, antidiabetic, anti‐inflammatory, antimalarial, analgesic, antihypertensive, antihyperglycaemic, antiangiogenic, antiulcer, immunomodulatory, antifungal, antipyretic, antihaemorrhoid, hepatoprotective, diuretic and antiallergy	[76, 123–125]
*Bothriocline longipes* (Oliv. & Hiern) N.E.Br.	Flavonoids and glycosides	Antibacterial	[126]
*Brachylaena huillensis* O.Hoffm.	Coumarins, essential oils, ketoalcohols and ketoaldehyde sesquiterpenes, sterols, tannins and triterpenes	Antibacterial, antifungal, antioxidant and antiprotozoal	[127, 128]
*Chromolaena odorata* (L.) R.M.King & H.Rob.	Anthraquinones, flavonoids, steroids, phenols, tannins, cardiac glycosides, saponins, terpenoids and essential oils	Anthelminthic, antimicrobial, anticancer, antibacterial, antifungal, analgesic, anti‐inflammatory, antidiarrhoeal, antioxidant, anticonvulsant, antiprotozoal, antipyretic, antispasmodic and cytotoxicity	[129–131]
*Chrysanthellum indicum* DC.	Flavonoids, terpenoids, phenylpropanoids and phenolics	Anti‐inflammatory, antioxidation, antipathogenic, anticancer, immune regulation and hepatoprotective	[132]
*Crassocephalum crepidioides* (Benth.) S.Moore	Alkaloids, flavonoids and phenolics	Antibacterial, wound healing, antidiabetic, anti‐inflammatory and antioxidant	[133]
*Crassocephalum vitellinum* (Benth.) S.Moore	Tannins, saponins, flavonoids and terpenoids	Antimicrobial and antiulcer	[134]
*Cyanthillium cinereum* (L.) H.Rob.	Sterols, triterpenoids, tannins, flavonoids, lactones and sesquiterpenes	Antibacterial, anticancer, analgesic, anti‐inflammatory and antiarthritic	[135, 136]
*Dichrocephala integrifolia* (L.f.) Kuntze	Tannins, flavonoids, alkaloids, terpenoids, phenols, glycosides and saponins	Antimicrobial, antiviral, cytotoxicity and anti‐inflammatory	[137, 138]
*Dicoma anomala* Sond.	Flavonoids, acetylenic compounds, saponins, diterpenes, phenols, phytosterols, tannins, sesquiterpenes and triterpenes	Antiviral, antibacterial, anthelminthic, antispasmodic, antiplasmodial, analgesic, anti‐inflammatory, anticancer, hepatoprotective, antihyperglycaemic and wound healing	[139, 140]
*Emilia coccinea* (Sims) G.Don	Flavonoids, alkaloids, tannins, saponins, oxalates, phenols and terpenoids	Antifungal, antioxidant, antidiarrhoeal and antimicrobial	[141–143]
*Erigeron bonariensis* L.	Flavonoids and terpenoids	Antioxidant, antibacterial, anti‐inflammatory, diuretic and antidiarrhoeal	[144]
*Erigeron canadensis* L.	Saponins, diterpenoids, terpenoids, glycosides, tannins, anthraquinones, steroids and flavonoids	Antimicrobial, antioxidant, anticoagulant, anti‐inflammatory, anticancer and mutagenic gastric protective	[145]
*Galinsoga parviflora* Cav.	Flavonoids, saponins, terpenoids and tannins	Antibacterial, antifungal, antidiabetic and antioxidant	[146]
*Gymnanthemum coloratum* (Willd.) H.Rob. & B.Kahn	Flavonoids, glycosides, saponins, steroids, tannins, glaucolides, lactones, amino acids, essential oils, alkaloids, anthocyanins, cardenolides, coumarins, leucoanthocyanins, phenols, quinones, reducing sugars, terpenoids and triterpenes	Anthelmintic, antimalarial, antioxidant, antidiabetic, antimicrobial, antiblastocystis, anti‐inflammatory, antisickling, insecticidal, larvicidal, antiplasmodial, antiproliferative, antitoxoplasma, hypoglycaemic and cytotoxicity	[147, 148]
*Gymnanthemum myrianthum* (Hook.f.) H.Rob.	Alkaloids, flavonoids, tannins, terpenoids, carotenoids and coumarin	Antimicrobial	[149]
*Helianthus annuus* L.	Phenolic acids, flavonoids and tocopherols	Antioxidant, antibacterial, antiviral, antiallergic, antithrombotic, vasodilatory, antimicrobial, antidiabetic, antihypertensive, anti‐inflammatory and wound healing	[150]
*Helichrysum foetidum* (L.) Moench	Flavonoids, terpenoids, foetidumins, chalcopyrones, steroids and glycerols	Antileishmanial and antiplasmodial, cytotoxicity, antiproliferative, antibacterial, antifungal, antiviral, anti‐HIV, antimalarial, antiulcerogenic, antityrosinase, anti‐inflammatory and antioxidant	[151, 152]
*Helichrysum odoratissimum* (L.) Sweet	Chalcones, helichrysetin and flavonoids	Antibacterial, antimycobacterial, antifungal, anti‐inflammatory, antioxidant, hepatoprotection, hypoglycaemic and cytotoxicity	[153]
*Launaea cornuta* (Hochst. ex Oliv. & Hiern) C.Jeffrey	Flavonoids	Antioxidant, anti‐inflammatory, antibacterial, anti‐proliferative and cytotoxic	[154, 155]
*Linzia glabra* Steetz	Alkaloids, flavonoids, saponins, terpenoids, sesquiterpene lactones, steroids, tannins, glycosides, phenols and quinones	Antifungal, antiviral, antibacterial and antihypertensive	[156, 157]
*Lipotriche scandens* (Schumach. & Thonn.) Orchard	Phenols, alkaloids, tannins, cardiac glycosides, saponins and steroids	Antibacterial, antioxidant, hepatoprotective, anti‐inflammatory and antitumor	[158–160]
*Microglossa pyrifolia* (Lam.) Kuntze	Flavonoids, alkaloids, glycosides, phenols, saponins, steroids and terpenoids	Antibacterial, antioxidant and antifungal	[80, 161]
*Montanoa hibiscifolia* Benth.	Flavonoids and terpenoids	Anti‐fertility, antimicrobial and antitumor	[162]
*Pluchea dioscoridis* (L.) DC.	Flavonoids, tannins, phenolic acids, phenylpropanoids, chalcones, eudesmane‐type sesquiterpenoids, monoterpenes, lignan glycosides and triterpenoids	Antioxidant, hepatoprotective, cytotoxic, antiulcer, antipyretic, antibacterial, antifungal, antiviral, antinociceptive, antiamoebic, antivenin, antidyslipidaemic and anti‐inflammatory	[163, 164]
*Pseudoconyza viscosa* (Mill.) D’Arcy	Phenolics and flavonoids	Antibacterial	[165]
*Psiadia punctulata* Vatke	Flavonoids, coumarins, terpenoids and phenylpropanoids	Antimicrobial, antiviral, anti‐inflammatory, antiplasmodial and antileishmanial	[166]
*Silybum marianum* (L.) Gaertn.			
*Solanecio angulatus* (Vahl) C.Jeffrey	Alkaloids, coumarins, flavonoids, quinones, glycosides, phenolics, steroids and tannins	Antitrypanosomal, anticancer, cytotoxicity and hepatoprotective	[167–169]
*Solanecio mannii* (Hook.f.) C.Jeffrey	Alkaloids and coumarins	Anthelminthic, antidiarrhoeal, anticancer, antivenin and cytotoxicity	[170–172]
*Tagetes minuta* L.	Essential oils, flavonoids, terpenoids, thiophenes, sterols and phenolics	Antibacterial, antifungal, anticancer, antiviral, antimalarial, antioxidant, antimicrobial, antidiabetic and antidepressant	[173–175]
*Tithonia diversifolia* (Hemsl.) A.Gray	Cardiac glycosides, flavonoids, alkaloids, anthraquinones, saponins, chlorogenic acid, sesquiterpenes and lactones	Antibacterial and antimicrobial	[176–179]
*Tridax procumbens* L.	Flavonoids, saponins, essential oils and terpenoids	Antimicrobial, antioxidant, anticancer, anti‐inflammatory and wound healing	[180]
*Vernonia amygdalina* Del.	Tannins, saponins, flavonoids, cyanogenic glycosides, alkaloids, terpenes, coumarins, anthraquinones, steroids, lignans, xanthones, edotides, sesquiterpenes, phenols, oxalates, phytates and phlobatannins	Anticancer, antidiabetic, antimalarial, anti‐inflammatory, cathartic, antimicrobial, hepatoprotective, antioxidant, chemoprotective, cytotoxic, analgesic, antipyretic, anthelmintic and antihypolipidaemic	[181, 182]

The production of secondary metabolites in Asteraceae MPs is profoundly shaped by ecological dynamics, particularly plant–microbe interactions and climate‐induced stressors. As emphasised by Hayat et al. [187] and Petrova et al. [188], these environmental factors act as regulatory signals that modulate biosynthetic pathways, thereby influencing both plant resilience and the therapeutic efficacy of their bioactive compounds. For instance, symbiotic associations with rhizosphere microbes can enhance the synthesis of flavonoids, terpenoids and alkaloids, whilst pathogenic challenges often trigger defensive metabolite cascades that simultaneously expand pharmacological diversity. Similarly, abiotic stressors such as drought, elevated temperatures or shifts in soil chemistry can upregulate stress‐responsive genes, leading to altered concentrations of compounds with antioxidant, anti‐inflammatory or antimicrobial properties. This perspective underscores that the pharmacological potential of Asteraceae MPs is inseparable from the ecological conditions under which they grow, suggesting that conservation strategies, cultivation practices and climate adaptation policies must account for these dynamic interactions to safeguard both biodiversity and medicinal value. In this way, ecological context is not merely a background variable but a fundamental determinant of therapeutic outcomes in plant‐derived remedies.

## 4. Conclusion

The Asteraceae family is a vital ethnomedicinal resource in Tanzania, with 73 species widely used for gastrointestinal, dermatological and pain‐related conditions. The dominance of herbs and the identification of flavonoids, terpenoids and phenolics as key bioactive compounds highlight the pharmacological diversity of the family, offering antibacterial, anti‐inflammatory, antioxidant, antitumor and antidiabetic effects. Besides validating traditional practices, these findings have significant implications for policy and practice. Firstly, they emphasise the need for national frameworks that integrate traditional medicine into primary healthcare systems, ensuring safe, affordable and culturally appropriate treatments. Secondly, they call for conservation strategies to protect MP biodiversity from overharvesting and environmental pressures. Finally, they stress the importance of research investment and capacity building to translate indigenous knowledge into evidence‐based medicine, aligned with Tanzania’s health priorities and SDGs. In conclusion, the Asteraceae family provides Tanzania with a dual opportunity: preserving cultural heritage whilst fostering scientific innovation. Embedding ethnomedicinal knowledge into governance, conservation and healthcare integration will strengthen resilience, improve access to affordable treatments and connect traditional medicine with modern biomedical research.

## Author Contributions

D.S.K. conceived, designed and conducted the study, collected and analysed the data and wrote the article.

## Funding

No funding was received for this manuscript.

## Conflicts of Interest

The author declares no conflicts of interest.

## Data Availability

The data that support the findings of this review are available from the corresponding author upon reasonable request.
